# A case of autoimmune pulmonary alveolar proteinosis in a patient with chronic nicotine vaping exposure

**DOI:** 10.1016/j.rmcr.2026.102475

**Published:** 2026-08-13

**Authors:** Jennifer L. Wong, Omar M. Pandhair, Cameron M. Long

**Affiliations:** a Internal Medicine, Providence St. Vincent Medical Center, Portland, OR, USA; b Pulmonology, Critical Care, and Sleep Medicine, The Oregon Clinic, Portland, OR, USA

## Abstract

Pulmonary alveolar proteinosis (PAP) is a rare diffuse lung disorder characterized by accumulation of lipoproteinaceous surfactant material within alveoli, leading to impaired gas exchange. While autoimmune PAP, mediated by granulocyte/macrophage colony-stimulating factor (GM-CSF) autoantibodies, represents the most common form, emerging environmental exposures have been implicated as potential triggers. Electronic cigarettes (e-cigarette) use, or vaping, has recently been reported in a limited number of cases as a possible contributing factor in the development of PAP.

We report a case of PAP in a young woman with a history of chronic vaping exposure, whose diagnostic evaluation confirmed the presence of circulating GM-CSF autoantibodies, supporting an autoimmune process linking the association between vaping and PAP.

## 1 Introduction

Pulmonary alveolar proteinosis (PAP), a rare diffuse lung disease, results from inappropriate accumulation of pulmonary alveolar surfactant. This accumulation is due to impaired clearance, from dysregulated granulocyte/macrophage colony-stimulating factor (GM-CSF) signaling [1]. Its incidence is approximately 0.2 cases per million, with a prevalence ranging from 4 to 40 cases per million [2]. Autoimmune PAP (aPAP) accounts for nearly 90% of cases, whereas the remaining 10% of cases are secondary to conditions such as hematologic malignancies, infections, or inhalational exposures to toxic particles [1,2].

E-cigarette or vaping-associated lung injury (EVALI) has emerged as a clinically significant pulmonary process linked to a spectrum of pathologies. However, its association with PAP remains exceedingly rare, with only a limited number of cases reported in the literature [3,4]. We present a unique case of autoimmune PAP in a young patient with a history of heavy nicotine vaping, highlighting a potential relationship between chronic inhalational exposure and the development of GM-CSF autoimmunity.

## 2 Case presentation

A 28-year-old woman with mild, intermittent asthma and daily nicotine vaping use, presented to the emergency department with progressively worsening dyspnea on exertion. Eighteen months earlier, she began regularly using a nicotine vaporizer, up to fifteen times daily. On initial evaluation, examination revealed diffuse crackles on auscultation. A computed tomography (CT) scan of the chest revealed bilateral ground-glass changes and interlobular septal thickening consistent with a crazy paving pattern (Fig. 1A). The initial differential diagnosis included atypical pneumonia, acute interstitial lung disease, pneumonitis, EVALI, and pulmonary alveolar proteinosis. Her autoimmune workup involving serum antinuclear antibody, rheumatoid factor, and antineutrophilic cytoplasmic antibody panels were negative. Urgent bronchoscopy was performed with return of opaque, off-white fluid on bronchoalveolar lavage (BAL). While BAL bacterial, fungal, *Nocardia* cultures and GM-CSF Elisa assay returned negative, periodic acid-Schiff (PAS) staining was positive. She was treated with a five-day course of doxycycline for possible atypical pneumonia with gradual symptomatic improvement. She was advised to stop vaping and discharged on room air.

**Fig. 1a:**
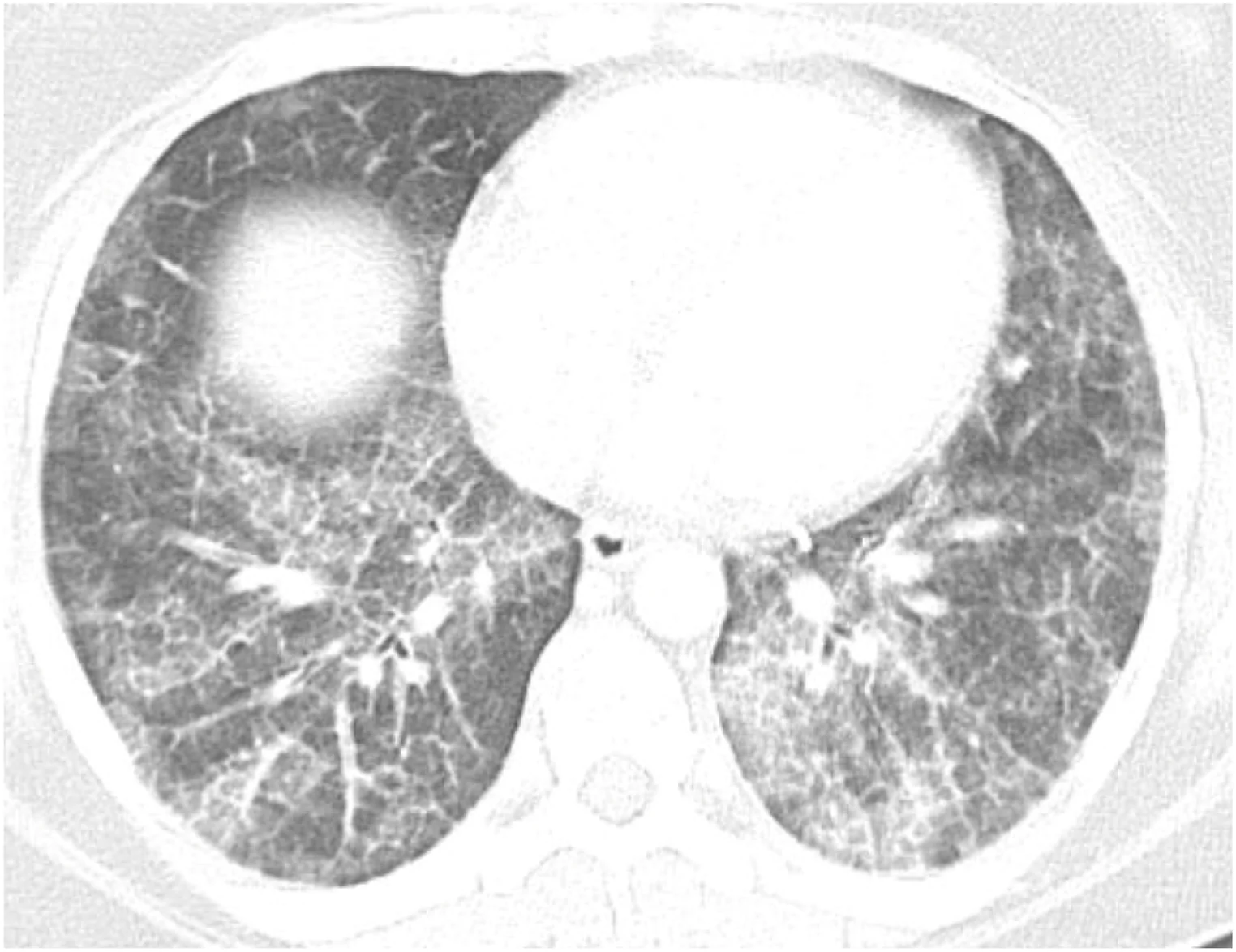
CT Chest with Contrast on initial admission demonstrating multifocal opacities and crazy paving pattern.

Three months later, she continued to vape up to five times daily and returned with worsening dyspnea and hypoxemia. A repeat CT chest showed worsening multifocal opacities and increased septal thickening (Fig. 1B). She underwent a repeat bronchoscopy with BAL and transbronchial biopsy. Pathology and cytology confirmed the diagnosis of PAP. Serum GM-CSF was low (Mayo Clinic Laboratories) and serum anti-GM-CSF autoantibodies (National Jewish Laboratories) returned elevated, consistent with autoimmune PAP. She was discharged on supplemental oxygen with exertion, motivated to stop vaping, and underwent a whole lung lavage two weeks later. This procedure involved a sequential lavage of each lung with warmed 0.9% normal saline (37 degrees Celsius), 1 L at time, and allowing gravity to drain. Drainage was aided by a manual chest percussion device and the initial effluent was densely milky in appearance. Her right lung was lavaged with 10 L of fluid and the left lung with 12 L. The lavage effluent progressively became more translucent (Fig. 2a, Fig. 2b).

**Fig. 1b:**
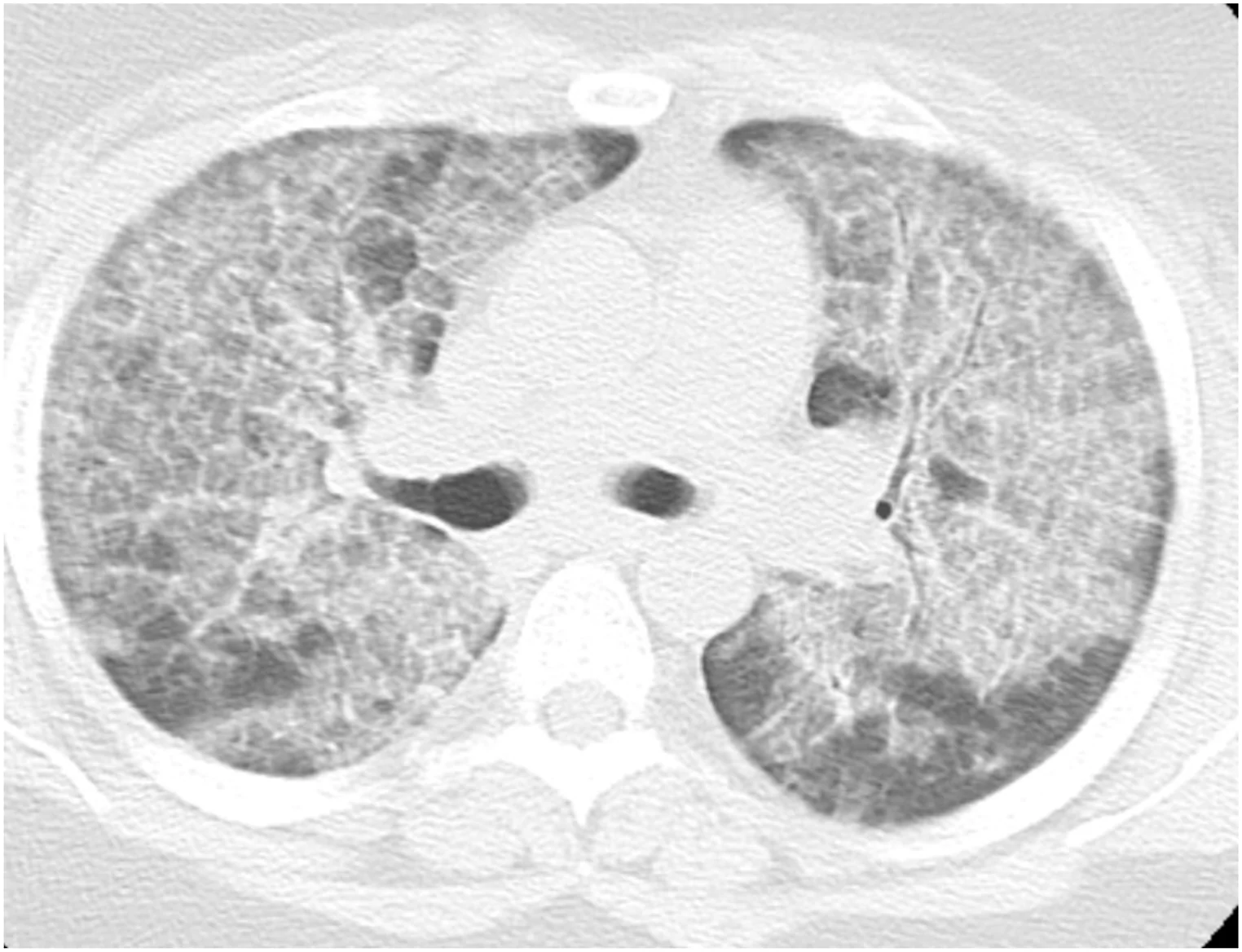
CT Chest without Contrast on subsequent admission showing increased multifocal opacities.

**Fig. 2a:**
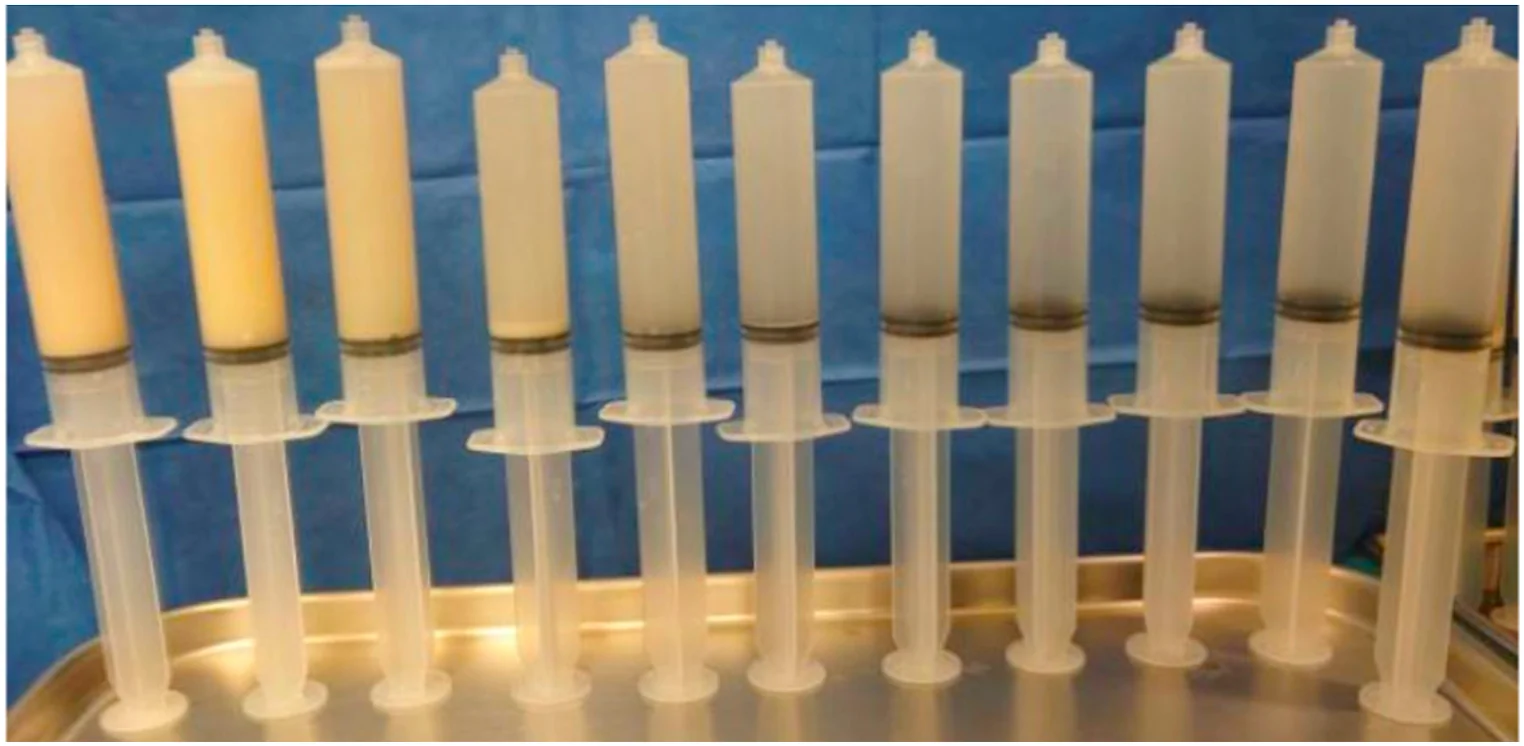
Right whole lung lavage return represented in 60 mL syringes for each liter (1 to 11.5) from left to right.

**Fig. 2b:**
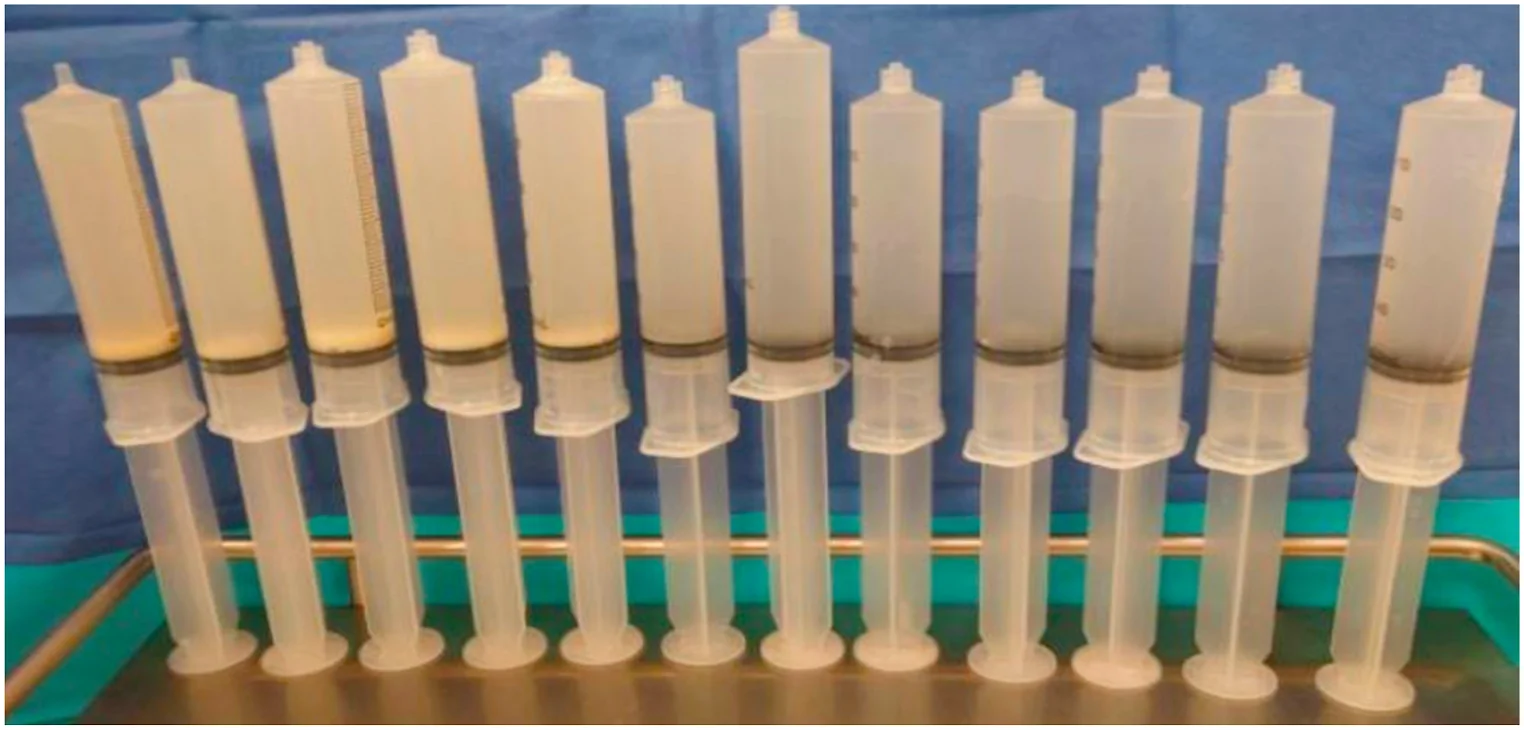
Left whole lung lavage return represented in 60 mL syringes for each liter (1 to 12) from left to right.

She was initiated on nebulized Sargramostim (Leukine) to take daily for one week, then off for one week, in a continuous alternating fashion for 28 weeks, then once a week going forward. Her pulmonary function tests initially revealed mild restriction (TLC 70%) and mildly reduced DLCO (70%) with significant bronchodilator response. Unfortunately, she developed exertional hypoxemia, and decline of lung function (TLC 61%, DLCO 57%), requiring a repeat whole lung lavage one year later with a total of 12 L lavaged on the right lung and 10 L on the left (Fig. 3a, Fig. 3b). After the lavage, she has been maintained on alternating weeks of Sargramostim daily, for which her exercise tolerance and pulmonary function testing have markedly improved (TLC 73%, DLCO 79%).

**Fig. 3a:**
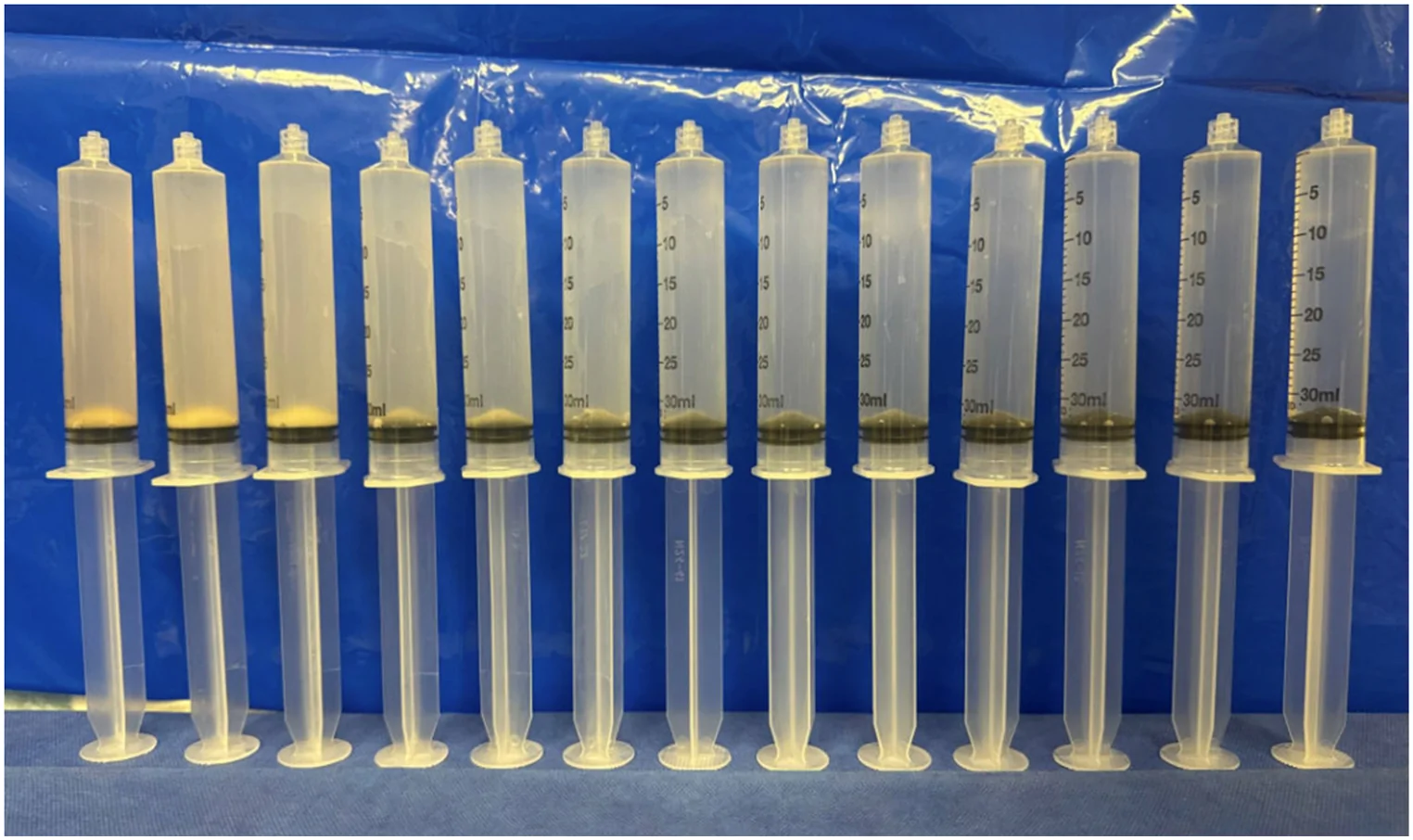
Right whole lung lavage return represented in 30 mL syringes for each liter (1 to 13) from left to right.

**Fig. 3b:**
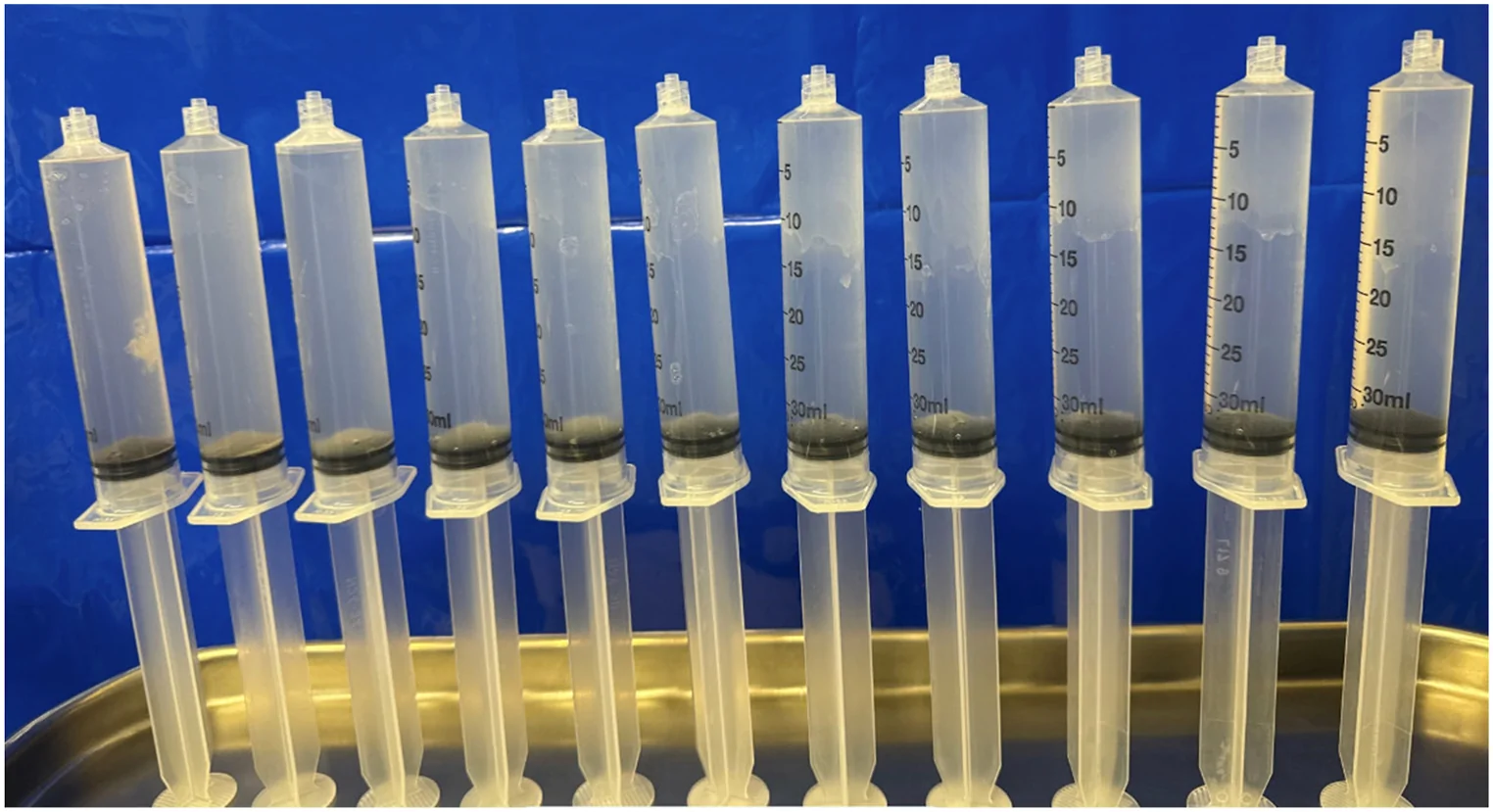
Left whole lung lavage return represented in 30 mL syringes for each liter (1 to 11) from left to right.

## 3 Discussion

Initially, our patient met all diagnostic criterion for EVALI and was advised to discontinue vaping [5]. However, her symptoms failed to resolve, and she was subsequently diagnosed with PAP. PAP has previously been reported in association with vaping exposure, however the pathogenesis still needs to be elucidated.

Two case reports have suggested that PAP may occur as a complication of EVALI. In both reports, serum anti-GM-CSF antibody testing (National Jewish Laboratories) was not performed; therefore, although one case was classified as secondary PAP and the other as aPAP, definitive diagnostic confirmation was not completed [3,4].

Israel, AK et al. 2020 proposed that vaping exposure functioned as a toxic inhalational injury causing direct macrophages dysfunction and damage, while Chua, TH et al. 2021 suggested that “vaping products … incite self-reactivity,” to trigger aPAP.

This case adds to the limited literature describing PAP in patients with a history of vaping exposure and supports the possibility that vaping exposure may coexist with or contribute to the development of autoimmune PAP in susceptible individuals. Interestingly, two cases of PAP were diagnosed among workers at indium processing facilities, which demonstrated positive serum GM-CSF autoantibodies in at least one affected individual, suggesting that toxic inhalation exposure can either unmask or precipitate autoimmune sensitization against GM-CSF [6]. In our patient, the diagnosis was ultimately established by positive serum anti-GM-CSF autoantibodies, confirming autoimmune PAP rather than secondary PAP alone. We hypothesize that vaping may act through a similar mechanism to induce PAP and suggest vaping-induced PAP be considered a separate entity from EVALI, though cessation of vaping should be recommended in both cases.

## 4 Conclusion

This case highlights the importance of anti-GM-CSF antibody testing in patients with presumed EVALI or exposure-associated PAP, as the distinction has important diagnostic and therapeutic implications. Furthermore, it raises the possibility that chronic inhalation exposure to vaping aerosols may act as an environmental trigger in susceptible individuals, potentially promoting immune dysregulation and the development of autoimmunity directed against anti-GM-CSF. Additional studies are needed to clarify the pathogenesis of vaping and autoimmune PAP and if there are specific host factors that lead to the development of the disease. Whole lung lavage and Nebulized Sargramostim are the only effective therapies to date in maintaining lung function.

## CRediT authorship contribution statement

**Jennifer L. Wong:** Writing – original draft, Writing – review & editing. **Omar M. Pandhair:** Writing – original draft, Writing – review & editing. **Cameron M. Long:** Writing – review & editing.

## Declaration of competing interests

The authors declare that they have no known competing financial interests or personal relationships that could have appeared to influence the work reported in this paper.
